# Longitudinal immune profiling reveals key myeloid signatures associated with COVID-19

**DOI:** 10.1126/sciimmunol.abd6197

**Published:** 2020-09-17

**Authors:** Elizabeth R. Mann, Madhvi Menon, Sean Blandin Knight, Joanne E. Konkel, Christopher Jagger, Tovah N. Shaw, Siddharth Krishnan, Magnus Rattray, Andrew Ustianowski, Nawar Diar Bakerly, Paul Dark, Graham Lord, Angela Simpson, Timothy Felton, Ling-Pei Ho, NIHR Respiratory TRC,, Marc Feldmann, CIRCO,, John R. Grainger, Tracy Hussell

**Affiliations:** 1 Lydia Becker Institute of Immunology and Inflammation, Division of Infection, Immunity & Respiratory Medicine, School of Biological Sciences, Faculty of Biology, Medicine and Health, University of Manchester, Manchester Academic Health Science Centre, Room 2.16, Core Technology Facility, 46 Grafton Street, Manchester, M13 9PL, UK.; 2 Maternal and Fetal Health Centre, Division of Developmental Biology, School of Medical Sciences, Faculty of Biology, Medicine and Health, The University of Manchester, 5th Floor St. Mary’s Hospital, Oxford Road, Manchester M13 9WL, UK.; 3 Respiratory Department, Salford Royal NHS Foundation Trust, Stott Lane, M6 8HD, UK.; 4 Division of Informatics, Imaging and Data Sciences, Faculty of Biology, Medicine and Health, University of Manchester, M13 9PL, UK.; 5 Regional Infectious Diseases Unit, North Manchester General Hospital, Manchester, UK.; 6 Intensive Care Department, Salford Royal NHS Foundation Trust, Stott Lane, M6 8HD, UK.; 7 Division of Infection, Immunity and Respiratory Medicine, Manchester NIHR BRC, Education and Research Centre, Wythenshawe Hospital, UK.; 8 MRC Human Immunology Unit, Weatherall Institute of Molecular Medicine, University of Oxford.; 9 Kennedy Institute of Rheumatology, Botnar Research Centre, Nuffield Department of Orthopedics, Rheumatology and Musculoskeletal Science, Windmill Rd, Headington, Oxford, OX3 7LD, UK

## Abstract

COVID-19 pathogenesis is associated with an exaggerated immune response. However, the specific cellular mediators and inflammatory components driving diverse clinical disease outcomes remain poorly understood. We undertook longitudinal immune profiling on both whole blood and peripheral blood mononuclear cells (PBMCs) of hospitalized patients during the peak of the COVID-19 pandemic in the UK. Here, we report key immune signatures present shortly after hospital admission that were associated with the severity of COVID-19. Immune signatures were related to shifts in neutrophil to T cell ratio, elevated serum IL-6, MCP-1 and IP-10, and most strikingly, modulation of CD14^+^ monocyte phenotype and function. Modified features of CD14^+^ monocytes included poor induction of the prostaglandin-producing enzyme, COX-2, as well as enhanced expression of the cell cycle marker K_i_-67. Longitudinal analysis revealed reversion of some immune features back to the healthy median level in patients with a good eventual outcome. These findings identify previously unappreciated alterations in the innate immune compartment of COVID-19 patients and lend support to the idea that therapeutic strategies targeting release of myeloid cells from bone marrow should be considered in this disease. Moreover, they demonstrate that features of an exaggerated immune response are present early after hospital admission suggesting immune-modulating therapies would be most beneficial at early timepoints.

## Teaser

Longitudinal analysis of the immune response in COVID-19 patients identifies a myeloid signature associated with severe disease.

## INTRODUCTION

Severe acute respiratory syndrome coronavirus 2 (SARS-CoV-2) infection can result in the clinical syndrome COVID-19 (*1*) that, to date, has resulted in over 20 million confirmed cases and in excess of 733,000 attributable deaths world-wide. As such, a large number of clinical trials have been established to evaluate anti-viral and immune modulatory strategies aimed at improving clinical outcome for this globally-devastating virus.

SARS-CoV-2 is a single stranded, positive sense RNA virus that enters cells via human angiotensin-converting enzyme 2 (ACE2) (*2*). Ordinarily, diverse immune mechanisms exist to detect every stage of viral replication and protect the host from viral challenge. Pattern recognition receptors of the innate immune system recognize viral antigen and virus-induced damage, increasing bone marrow hematopoiesis, the release of myeloid cells including neutrophils and monocytes, and the production of a plethora of cytokines and chemokines (*3*). If inflammatory mediator release is not controlled in duration and amplitude then “emergency haematopoiesis” leads to bystander tissue damage and a cytokine storm that manifests as organ dysfunction. Initial studies suggest cytokine storm occurs in COVID-19 (*4*). Indeed, neutrophilia and lymphopenia (resulting in an increased neutrophil to lymphocyte ratio), increased systemic interleukin-6 (IL-6) and C-reactive protein (CRP), correlate with incidence of intensive care admission and mortality (*5*). However, detailed understanding of cellular and molecular inflammatory mediators across the COVID-19 disease trajectory would support the development of better clinical interventions.

We carried out the Coronavirus Immune Response and Clinical Outcomes (CIRCO) study at four hospitals in Greater Manchester, UK, which was designed to examine the kinetics of the immune response in COVID-19 patients, as well as to identify early indicators of disease severity. Understanding the specific elements and kinetics of the immune response is critical to gain insight into immune phenotypes associated with disease progression, identify potential biomarkers that predict clinical outcomes and determine at which stage of the disease immune modulation may be most effective (*4*).

Here, by analyzing fresh blood samples immediately without prior storage we outline unappreciated immune abnormalities present within COVID-19 patients. Assessment of inflammatory mediators within the blood demonstrated these immune properties were most dysregulated in patients with severe COVID-19 prior to admission to intensive care, indicating immune modulating therapies should be considered early after admission. Furthermore, our study demonstrated profound alterations in the myeloid cells of COVID-19 patients. Our data demonstrate that monocytes from COVID-19 patients displayed elevated levels of the cell cycle marker K_i_-67 but reduced expression of the prostaglandin-generating enzyme COX-2, with both these features being predominant in severe COVID-19 patients. These findings not only identify possible immune biomarkers for patient stratification but potential mechanisms of immune dysfunction contributing to the immunopathology of COVID-19.

## RESULTS

### CIRCO patient clinical characteristics

**Fig. 1:**
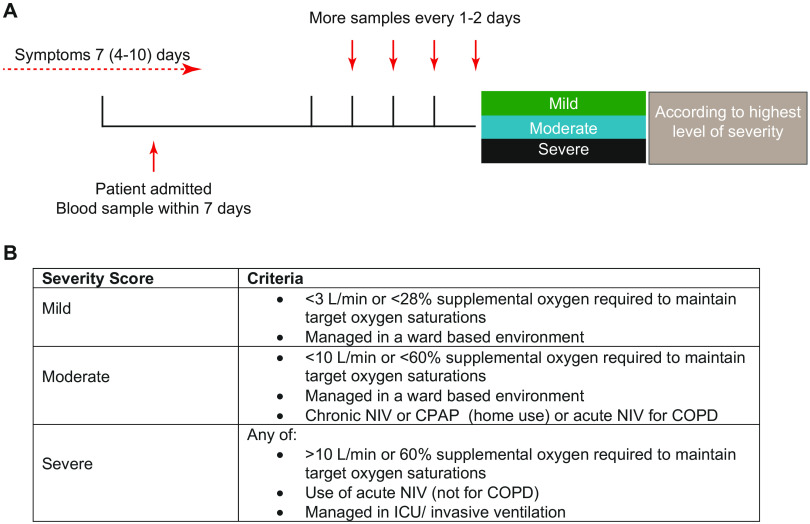
**Patient recruitment and categorization. (A)** Patients were recruited to the study as close to admission as possible and within 7 days. Peripheral blood samples were collected on recruitment and at intervals thereafter. Samples were analyzed immediately and results stratified based on their ultimate disease severity. **(B)** Criteria for patient stratification. NIV, non-invasive ventilation; CPAP, continuous positive airway pressure; ICU, intensive care unit.

In total, 73 patients were recruited and 49 were stratified for maximum disease severity (Fig. 1A). Six patients were excluded due to: an alternative diagnosis (2 patients); indeterminate imaging findings with negative result in the SARS-CoV-2 nasopharyngeal test (2 patients); or diagnosis of a confounding acute illness (2 patients). Two patients could not be stratified for disease severity due to insufficient clinical observation data and a further 16 were not stratified because recruitment occurred more than 7 days after admission. The median time from patient-reported symptom onset to hospital admission was 7 days. The overall median age was 61 and 63% were male. The most frequent co-morbidities were diabetes, ischemic heart disease, hypertension, asthma and chronic obstructive pulmonary disease (COPD) (Table 1). The majority (86%) of patients tested positive for SARS-CoV-2 via nasopharyngeal RT-PCR. In 14% of patients, symptoms and radiographic features were highly suggestive of COVID-19, but nasopharyngeal test was negative for the virus and thus a clinical diagnosis was made; these patients are clearly indicated in all graphs (white triangles). Patient disease severity was defined as mild (less than 28% FiO_2_), moderate (28-60% FiO_2_) or severe (above 60% FiO_2_, or admission to intensive care) (Fig. 1B). Death occurred in 50% of severe cases of COVID-19 and only one of the ten patients with severe disease was categorized as severe upon admission.

**Table 1:** Clinical characteristics. Data are listed as median (IQR) ^m^, where m is the number of missing data points, n (%), or n/N (%), where N is the total number with available data. PE, pulmonary embolism; AKI, Acute kidney injury. ^a^Admission observations. Representative participants from each severity cohort were used in cross-sectional or longitudinal analysis.

	**All patients (49)**	**Mild (18)**	**Moderate (21)**	**Severe (10)**
**Age**	61 (51–71)	61.5 (45–72.5)	59 (51–68)	66 (52–72.5)
**Sex**				
Male	31 (63.3%)	11 (61.1%)	13 (62%)	7 (70%)
Female	18 (36.7%)	7 (38.9%)	8 (38%)	3 (30%)
**BMI**	27.5 (24.9–30)^4^	27.1 (23.6–30)^1^	28.3 (25.7–30)^2^	26.5 (24.9–30.4)^1^
**Co-morbidity**				
Diabetes	8/49 (16.3%)	3/18 (16.7%)	2/21 (9.5%)	3/10 (30%)
Ischemic heart disease	5/49 (10.2%)	2/18 (11.1%)	1/21 (4.8%)	2/10 (20%)
Hypertension	14/49 (28.6%)	5/18 (27.8%)	7/21 (33.3%)	2/10 (20%)
Chronic Obstructive Pulmonary Disease	9/49 (18.4%)	4/18 (22.2%)	4/21 (19.1%)	1/10 (10%)
Asthma	5/49 (10.2%)	2/18 (11.1%)	3/21 (14.3%)	0/10 (0%)
Malignancy	3/49 (6.1%)	0/18 (0%)	1/21 (4.8%)	2/10, (20%)
**Presentation**				
Illness onset to admission (days)	7 (4–10)^4^	7 (4–8)^1^	7.5 (2.8–10.8)^3^	5.5 (4–9.5)
Dyspnea	29/41 (70.7%)	8/16 (50%)	14/16 (87.5%)	7/9 (77.8%)
Cough	30/41 (73.2%)	11/16 (68.8%)	12/16 (75%)	7/9 (77.8%)
Fever	28/41 (68.3%)	10/16 (62.5%)	9/16 (56.3%)	9/9 (100%)
Diarrhea/ Vomiting	14/41 (34.2%)	3/16 (18.8%)	7/16 (43.8%)	4/9 (44.4%)
Myalgia	10/40 (25%)	4/15 (26.7%)	3/16 (18.8%)	3/9 (33.3%)
Fatigue	10/38 (26.3%)	3/14 (21.4%)	5/15 (33.3%)	2/9 (22.2%)
Day recruited	2 (2–3)	3 (2–4.5)	2 (2–3)	2 (2–3)
Number of timepoints	2 (1–4)	2 (1 – 2)	3 (2–3)	4.5 (2–5)
Respiratory rate^a^	20 (18–25)^10^	20 (17–24)^5^	21 (18–26)^3^	21.5 (17.8–24)^2^
Temperature^a^	37.5 (36.9–38.4)^10^	37.1 (36.5–37.4)^5^	37.7 (37.1–38.8)^3^	38 (37.7–39.2)^2^
Systolic blood pressure^a^	125 (117–136)^10^	122 (117–126)^5^	126 (118–136)^3^	137.5 (114.5–156.3)^2^
**Chest Radiograph Findings**				
Bilateral opacification	41/47 (87.2%)	11/16 (68.8%)	20/21 (95.2%)	10/10 (100%)
Unilateral opacification	3/47 (6.4%)	2/16 (12.5%)	1/21 (4.8%)	0/10 (0%)
No abnormality	3/47 (6.4%)	3/16 (18.8%)	0/21 (0%)	0/10 (0%)
**COVID Nasopharyngeal Test**				
Positive	42 (86%)	15 (83.3%)	18 (85.7%)	9 (90%)
Negative	7 (14%)	3 (16.7%)	3 (14.3%)	1 (10%)
**Differential full blood count at admission**				
White blood cell count (x10^9^/L)	6.9 (5.7–9.8)	6.7 (4.7–7.5)	7.1 (6.3–10)	7.3 (5.7–10)
Lymphocytes (x10^9^/L)	1.1 (0.8–1.4)	1.2 (0.8–1.3)	1.3 (0.8–1.5)	0.9 (0.8–1.1)
Neutrophils (x10^9^/L)	5.1 (3.8–7.4)	4.7 (3.2–5.7)	5.3 (4.5–7.7)	6.3 (4.2–8.6)
Monocytes (x10^9^/L)	0.4 (0.2–0.7)	0.4 (0.2–0.6)	0.5 (0.3–0.7)	0.3 (0.2–0.5)
Platelets (x10^9^/L)	244 (188–367)^2^	241 (188–316)^2^	269 (195–366)	204 (155–412)
**Highest acute phase response/ Liver function tests**				
C-Reactive protein (CRP) (mg/L)	127 (75–226)	88 (38–166)	120 (75–201)	269 (244–296)
Alanine aminotransferase (U/L)	48 (27–87)^16^	57 (28–75)^9^	35 (25–79)^5^	49 (37–105)^2^
Alkaline phosphatase (U/L)	78 (63–96)^16^	79 (63–82)^9^	72 (63–90)^5^	110 (71–172)^2^
Bilirubin (μmol/L)	11 (7–15)^16^	10 (8–14)^9^	9 (7–13)^5^	13 (10–21)^2^
**Complications**				
PE	5/49 (10.2%)	1/18 (5.6%)	4/21 (19.1%)	0/10 (0%)
AKI	3/49 (6.1%)	0/18 (0%)	2/21 (9.5%)	1/10 (10%)
Mortality	6/49 (12.2%)	1/18 (5.6%)	0/21 (0%)	5/10 (50%)

### Broad shifts in the innate and adaptive immune compartments in COVID-19 patients

Based on blood cell counts by the hospital laboratory at admission, no significant differences in total white blood cells, neutrophils, monocytes or lymphocytes were observed between groups of COVID-19 patients that went on to progress to mild, moderate or severe disease (Fig. S1A). However, as reported previously (*6*, *7*), a trend was evident toward a higher neutrophil to lymphocyte ratio (NLR) at hospital admission in those patients whose outcome eventually was severe (Fig. S1B). This suggested that a more in-depth immune profiling could aid in patient stratification prior to escalation of the disease.

**Fig. 2:**
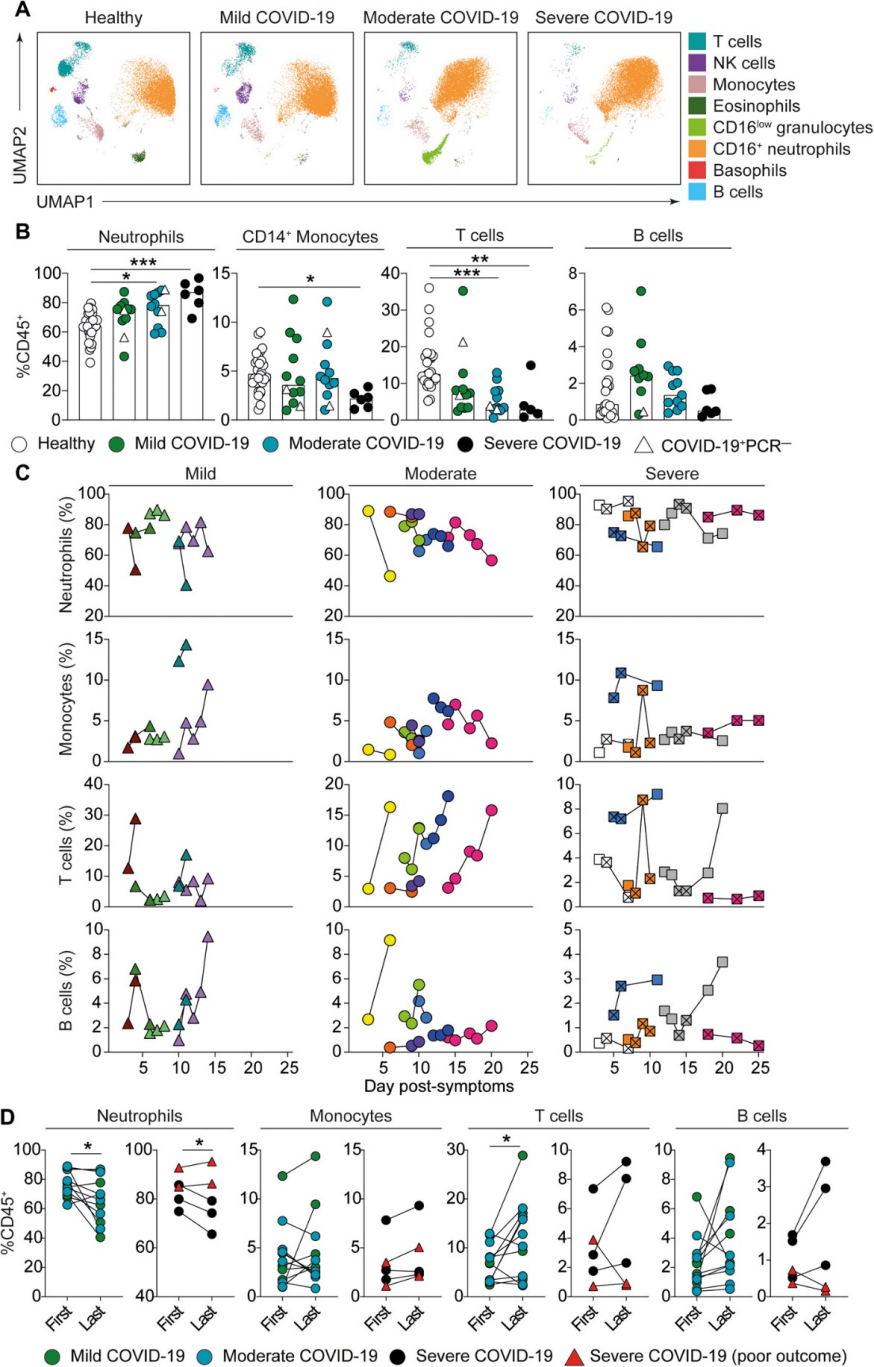
**Whole blood immune profile of COVID-19 patients. (A)** Uniform Manifold Approximation and Projection (UMAP) of flow cytometry panel broadly visualizing white cells in whole blood. Representative images for healthy individuals, mild, moderate and severe patients are shown. Key indicates cells identified on the image. **(B)** Graphs show neutrophil (CD16^+^CD11b^hi^), CD14^+^ monocyte, CD3^+^ T cell, and CD19^+^ B cell frequencies in whole blood samples of healthy individuals (n=28) and recruitment samples from COVID-19 patients with mild (n=12), moderate (n=13) and severe (n=6) disease. **(C)** Longitudinal time course of (top row) neutrophils (CD16^+^CD11b^hi^), (2^nd^ row) CD14^+^ monocytes, (3^rd^ row) CD3^+^ T cells and (bottom row) B cells segregated by disease severity. Individual patients are shown as different colors and shapes with lines connecting data from the same patient. Crossed squares for severe patients are time points in intensive care unit (ICU). X axis values represent the number of days since reported onset of symptoms. **(D)** Graphs showing frequencies of neutrophils (CD16^+^CD11b^hi^), monocytes, T cells and B cells at the first and last time points in (left) mild/moderate patients (green and blue circles) and (right) severe patients (black circles). Red triangles represent severe patients that had poor outcome (deceased or long-term ICU) and are not included in the statistical test. Graphs show individual patient data with the bar representing median values. In all graphs, open triangles represent SARS-CoV-2 PCR negative patients. Kruskal Wallis with Dunn’s post-hoc test; 2B Neutrophils, T cells and B cells. One-way ANOVA with Holm-Sidak post-hoc test: 2B Monocytes. Paired *t*-test; 2D all except monocyte graph detailing mild and moderate patients which was tested using Wilcoxon matched-pairs signed rank test. (*P<0.05, **P<0.01, ***P<0.001, ****P<0.0001).

Thus, we further explored alterations in the innate and adaptive immune compartments using high dimensional flow cytometry on white blood cells from freshly lysed whole blood (see Fig. S1C for gating strategy). Initially, we examined the first blood sample taken at the time of patient recruitment to the study (this was typically 2-3 days after hospital admission and was not greater than 7 days). At this recruitment time point, alterations to the characteristics and relative abundance of diverse immune cell types was observed. Uniform manifold approximation and projection (UMAP) visualization outlined alterations between patients and healthy controls in the characteristics of neutrophils and monocytes, dramatic increases in the frequency of neutrophils and decreased T cells, B cells and basophils. Cellular changes were exaggerated with disease severity (Fig. 2A). In a subset of infected individuals CD16^low^ granulocytes were present (Fig. 2A); these cells can be associated with altered immune cell output from the bone marrow (*8*). This global picture of alterations to innate and adaptive immune cells was confirmed by manual flow cytometric gating (Fig. 2B and Fig. S1, C and D). In addition to these alterations, examining cell frequencies within isolated peripheral blood mononuclear cells (PBMCs) revealed a decrease in the frequency of plasmacytoid dendritic cells (pDCs) in COVID-19 patients, that was enhanced with elevated disease severity (Fig. S1E). There were no changes observed in frequencies of CD56^+^ NK cells (Fig. S1E).

Given the dramatic alterations in neutrophil and T cell frequencies at the time of recruitment (Fig. 2B), we next examined their profile longitudinally over the course of hospitalization. To do this we used the first day of patient-reported symptom onset as a common reference point to align patient disease trajectories. This revealed that in the majority of patients, irrespective of final severity, neutrophil frequencies, although initially extremely high, decreased prior to hospital discharge while T cell frequencies reciprocally increased (Fig. 2C and 2D). In contrast, CD14^+^ monocytes and B cells showed no obvious trends during the hospital stay (Fig. 2C and D). These data highlight the importance of examining neutrophil to lymphocyte ratio in COVID-19 patients (*6*, *7*), but, along with other studies (*9*), indicate that assessment of neutrophil to T cell ratio may provide a more stringent disease insight. Notably, in two severe patients with poor outcome, T cell frequencies were extremely low and neutrophil frequencies high even after entry into an intensive care unit (ICU) (Fig. 2C; white and pink crossed squares and Fig. 2D, red triangles); indicating that rebalancing of neutrophil to T cell ratio is crucial to recovery.

### Defined soluble mediators are associated with severe disease

Broad changes in circulating immune cells in other viral infections are associated with alterations to circulating inflammatory mediators, such as cytokines and chemokines. These are potent modifiers of bone marrow output, immune cell survival and cell-recruitment to the inflamed lung. We used multiplex bead array to assess soluble inflammatory mediators in serum from patients at recruitment to the study. Of the 13 mediators analyzed in serum IL-6, IL-10, monocyte-chemoattractant protein-1 (MCP-1) and interferon gamma-induced protein 10 (IP-10) were significantly increased in COVID-19 patients and tracked with disease severity (Fig. S2A). No significant changes in other cytokines or chemokines measured, including IFN-γ, IL-1β, IL-8 and TNF-α were observed in COVID-19 patients (Fig. S2B).

Interestingly, longitudinal analysis (examined as above from the day of reported disease onset) of IL-6, MCP-1 and IP-10 in mild and severe patients revealed that the highest levels of these cytokines and chemokines occurred early in the disease trajectory at recruitment to the study (Fig. S2C). Indeed, there was a significant decrease in IL-6 and IP-10 in patients upon recovery (Fig. S2D). There was a dramatic reduction in IL-6, IP-10 and MCP-1 upon admission of severe patients into ICU from the ward (Fig. S2E), although this finding is based on just 3 patients. This may be due to the treatment modalities employed in intensive care, such as sedation, that can have immunomodulatory effects (*10*), and will be important to investigate further. Interestingly, the patient whose health declined rapidly following admission, and ultimately died from the disease, displayed a dramatic rebound in IL-6 and MCP-1 levels after 2 days on ICU (Fig. S2, D and E; red triangles).

### Activation of adaptive immune cells in COVID-19 patients

**Fig. 3:**
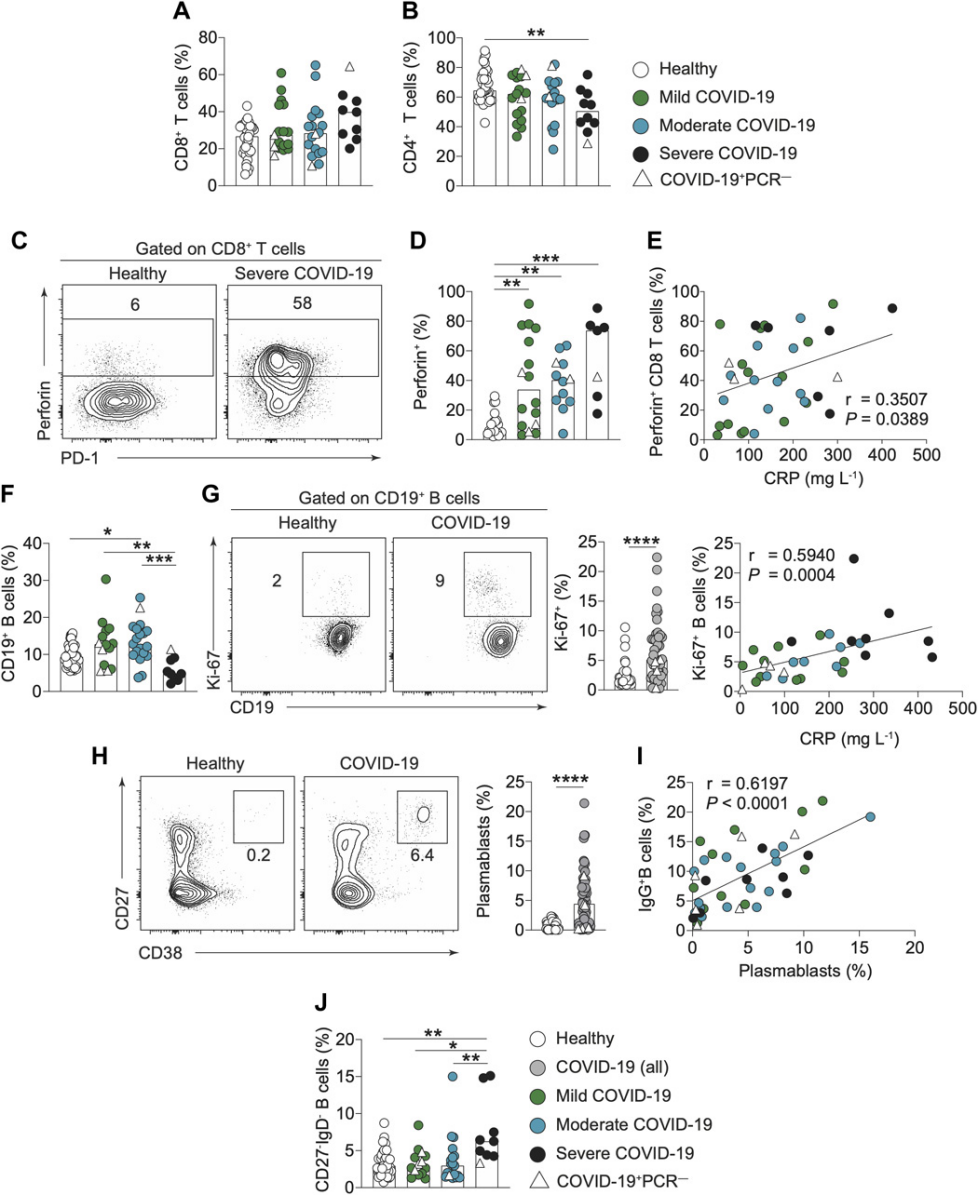
**Altered phenotype of T and B cells in COVID-19 patients. (A,B)** Graphs show frequencies of **(A)** CD8^+^ and **(B)** CD4^+^ T cells in freshly isolated PBMCs of healthy individuals (n=36) and recruitment samples from COVID-19 patients with mild (n=17), moderate (n=18) and severe (n=9-10) disease. **(C,D)** Representative flow cytometry plots and graph showing frequency of CD8^+^ T cells which are positive for perforin in healthy individuals (n=21 and COVID-19 patients with mild (n=16), moderate (n=12) and severe (n=7) disease. **(E)** Graph showing correlation of perforin^+^ CD8^+^ T cell frequency with C-reactive protein (CRP) in COVID-19 patients. **(F)** Graphs show frequencies of CD19^+^ B cells in freshly isolated PBMCs of healthy individuals (n=43) and recruitment samples from COVID-19 patients with mild (n=14), moderate (n=19) and severe (n=9) disease. **(G)** Representative flow cytometry plots and cumulative data show K_i_-67 expression by B cells in healthy individuals (n=39) and COVID-19 patients (n=45). Correlation graph shows correlation of K_i_-67^+^ B cells with C-reactive protein (CRP). **(H)** Representative flow cytometry plots and cumulative data show frequency of CD27^hi^CD38^hi^ plasmablasts in healthy individuals (n=42) and COVID-19 patients (n=66). **(I)** Correlation graph shows correlation of plasmablasts and IgG^+^ B cell frequencies. **(J)** Graph shows frequencies of double negative (CD27^-^IgD^-^) B cells in freshly prepared PBMC of healthy individuals (n=42) and recruitment samples from COVID-19 patients with mild (n=14), moderate (n=19) and severe (n=9) disease. Graphs show individual patient data with the bar representing median values. In all graphs, open triangles represent SARS-CoV-2 PCR negative patients. Mann-Whitney U test; 3G, 3H. Kruskal Wallis with Dunn’s post-hoc test; 3A, 3D, 3F, 3J. One-way ANOVA with Holm-Sidak post-hoc test: 3B. Spearman ranked coefficient correlation test; 3E, 3G, 3I. (*P<0.05, **P<0.01, ***P<0.001, ****P<0.0001).

To build on our basic assessment of cell populations outlined in Fig. 2, we next investigated alterations to specific T and B cell populations by flow cytometrically analyzing isolated peripheral blood mononuclear cells (PBMCs). Within the T cell compartment, we noted no dramatic alterations in CD4^+^ or CD8^+^ T cell frequencies (Fig. 3A and B). However, a slight decrease in CD4^+^ T cells was observed in severe COVID-19 patients (Fig. 3B). Both T cell subsets showed signs of activation in COVID-19 patients and this was more apparent in CD8^+^ T cells. Of note, the degree of T cell activation did not track with disease severity and was highly variable amongst patients (Fig. S3, A to D). Despite this, COVID-19 patients exhibited decreased frequencies of naive but elevated frequencies of effector TEMRA and HLA-DR^+^CD38^+^ CD8^+^ T cells (Fig. S3, A to C). CD8^+^ T cell subsets remained remarkably stable over the hospitalized disease course (Fig. S3E).

Interestingly, in 34/43 COVID-19 patients, higher perforin expression was observed in CD8^+^ T cells compared to healthy individuals (Fig. 3C and Fig. S3F), implying CD8^+^ T cells in COVID-19 patients had activated a cytotoxic program. Perforin expression in CD8^+^ T cells did not significantly track with disease severity (Fig. 3D), but a positive correlation was observed between the frequency of perforin^+^CD8^+^ T cells and clinical measurements of the inflammatory marker C-reactive protein (CRP) (Fig. 3E). This indicates increased frequencies of circulating perforin^+^ CD8^+^ T cells are more prevalent in highly inflamed patients. However, perforin^+^CD8^+^ T cells were found to increase over time in mild and most moderate COVID-19 patients, with highest levels immediately prior to discharge (Fig. S3, G to H), suggesting the higher frequencies seen in severe patients are not necessarily detrimental. This enhancement over time in mild and moderate patients suggests the higher frequencies seen in severe patients are not necessarily detrimental. Overall, these data demonstrate heterogeneous T cell activation in COVID-19 patients, but a consistent cytotoxic profile in the CD8^+^ T cell compartment.

Similar to the trend in whole blood (Fig. 2B), B cell frequency was reduced in PBMCs of COVID-19 patients. Decreases were particularly striking in severe patients compared to those with mild and moderate disease (Fig. 3F) and persisted with time (Fig. S4A). Although reduced in frequency, B cells displayed increased expression of K_i_-67 (indicative of proliferation), which positively correlated with CRP levels (Fig. 3G). When examining B cell subsets, we observed an expansion of antibody-secreting plasmablasts (CD27^hi^CD38^hi^CD24^–^), that positively correlated with IgG expression by B cells (Fig. 3H and I). Further, we observed a decrease in unswitched memory (CD27^+^IgD^+^IgM^+^) B cells but no global differences in frequencies of other B cell subsets (Fig. S4B). Of note, the differences in B cell subsets did not track with disease severity (Fig. S4C). The only subpopulation of B cells dramatically expanded in patients with severe COVID-19, compared to patients with mild and moderate disease, was double negative (DN) B cells (CD27^–^IgD^–^) (Fig. 3J). This subset was relatively stable throughout patient hospitalization and associated with a worse disease trajectory (Fig. S4D). DN B cells have previously been associated with an exhausted phenotype in patients with HIV (*11*), suggesting that patients with severe COVID-19 may have an impaired capacity to generate an effective B cell response.

### Altered monocyte phenotype and function is a feature of COVID-19

**Fig. 4:**
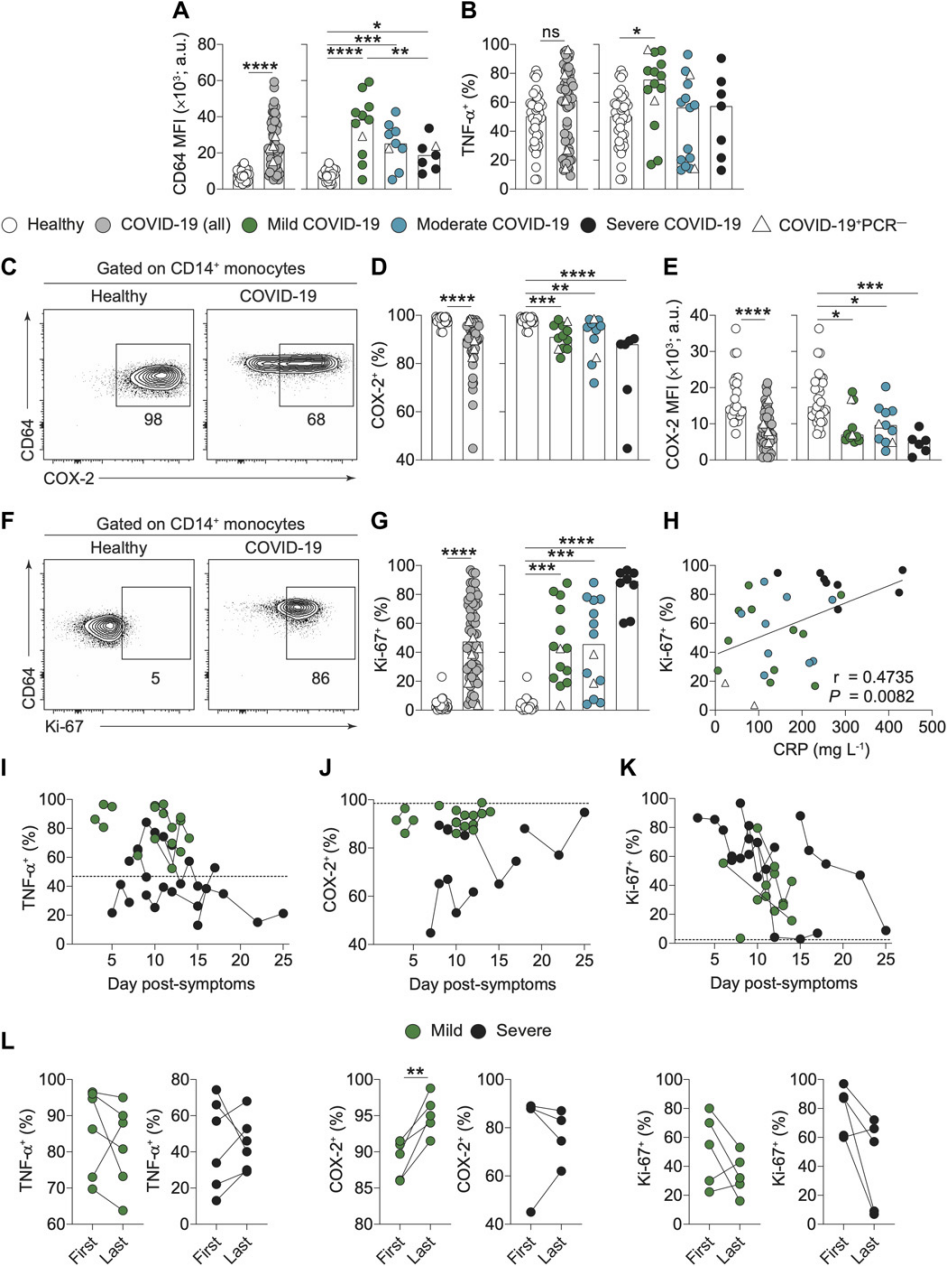
**Dysregulation of circulating monocytes in COVID-19. (A)** Graphs show levels of CD64 expression as assessed by mean fluorescence intensity (MFI) on CD14^+^ classical monocytes in freshly prepared PBMC of healthy individuals (n=25) and recruitment samples from all COVID-19 patients (n=58). COVID-19 patients were also stratified into mild (n=12), moderate (n=10) and severe (n=8) disease. **(B)** Graphs show frequencies of TNF-ɑ^+^ CD14^+^ monocytes following LPS stimulation of freshly prepared PBMC from healthy individuals (n=41) and COVID-19 patients (n=59). COVID-19 patients were also stratified into mild (n=14), moderate (n=15) and severe (n=7) disease. **(C)** Representative FACS plots demonstrating intracellular COX2 expression by CD14^+^ monocytes from healthy individuals and COVID-19 patients. **(D, E)** Graphs showing **(D)** frequencies of COX-2^+^ CD14^+^ monocytes and **(E)** COX-2 expression level as determined by MFI in CD14^+^ monocytes following LPS stimulation of freshly prepared PBMC from healthy individuals (n=33) and total COVID-19 patients (n=51). COVID-19 patients were also stratified into mild (n=12), moderate (n=11) and severe (n=6) disease. **(F)** Representative FACS plots demonstrating intracellular K_i_-67 staining by CD14^+^ monocytes. **(G)** Graphs show frequencies of K_i_-67^+^ CD14^+^ monocytes following LPS stimulation of freshly prepared PBMC from healthy individuals (n=37) and total COVID-19 patients (n=60). COVID-19 patients were also stratified into mild (n=14), moderate (n=14) and severe (n=8) disease. **(H)** Correlation of K_i_-67 (% of monocytes expressing K_i_-67) with CRP in COVID-19 patients. **(I-K)** Longitudinal time course of frequencies of CD14^+^ monocytes that are positive for **(I)** TNF-α, **(J)** COX2 and **(K)** K_i_-67 following LPS stimulation in mild (green shapes, n=6-7) and severe (black shapes, n=4-6) COVID-19 patients with lines connecting data from the same patient. On all graphs x axis values represent the number of days since onset of symptoms and the dotted line represents the median value from healthy individuals. **(L)** Graphs showing frequencies of monocytes which are TNF-α^+^, COX-2 and K_i_-67^+^ following LPS stimulation at the first and last time points in (left) mild patients (green circles) and (right) severe patients (black circles). Graphs show individual patient data with the bar representing median values. In all graphs, open triangles represent SARS-CoV-2 PCR negative patients. Mann-Whitney U test; 4A, 4B. 4D, 4E, 4G. Kruskal Wallis with Dunn’s post-hoc test; 4B, 4D, 4E, 4G. One-way ANOVA with Holm-Sidak post-hoc test:.4A. Spearman ranked coefficient correlation test; 4H. Paired *t*-test; 4L. (*P<0.05, **P<0.01, ***P<0.001, ****P<0.0001).

COVID-19 research to date has primarily focused on T and B cells, although recent publications have highlighted alterations to monocyte phenotype (*12*). Monocytes can contribute significantly to inflammatory disease directly or via differentiation to macrophages and dendritic cells (*13*, *14*). When released into the blood stream, monocytes will be affected by circulating cytokines and chemokines, including MCP-1, which we define as raised early in COVID-19 sera (Fig. S2A). In COVID-19 patients, we observed an expansion of intermediate CD14^+^CD16^+^ monocytes that tended to be highest in patients with a mild disease outcome (Fig. S5, A and B). Enhanced expression of CD64, the high affinity Fc receptor for monomeric IgG (FcγRI), was apparent on classical CD14^+^ monocytes (Fig. 4A) and again was most evident in mild disease.

We next examined monocyte activation by stimulating with lipopolysaccharide (LPS); stimulation frequencies of viable cells were high (greater than 90%) and similar in COVID-19 patients and healthy controls. Following stratification for final disease severity, TNF-α was enhanced in patients with mild disease (Fig. 4B and Fig. S5C). In contrast, IL-1β production was lower in monocytes from COVID-19 patients compared to monocytes from healthy individuals (Fig. S5D), although this was not related to disease severity. These data highlight that monocytes from COVID-19 patients exhibit a modified cytokine profile upon activation. As well as cytokines, monocytes are major producers of lipid mediators, such as prostaglandins (*15*) and so we also examined cyclooxygenase-2 (COX-2) expression (a rate-limiting enzyme in prostaglandin synthesis). Notably, in LPS-stimulated monocytes a reduction in COX-2 was evident in all COVID-19 patients and was most apparent in those with severe disease (Fig. 4, C to E). Accordingly, expression of COX-2 in stimulated monocytes was inversely correlated to systemic levels of the cytokine MCP-1 (Fig. S5E), which were highest in severe COVID-19 patients (Fig. S2A).

One possible reason that monocytes in COVID-19 patients display altered functionality in the periphery is due to inflammation-induced emergency myelopoiesis (*3*). This process occurs during infection where hematopoietic stem cells and myeloid progenitors expand in the bone marrow in order to provide more cells to combat viral infection. However, if egress is too fast then monocytes exit in an altered state. For example, unusually high expression of the cell cycle marker K_i_-67 is observed in peripheral monocytes during H1N1 influenza (*16*) and Ebola virus (*17*) infection. We therefore, investigated expression of the proliferation marker K_i_-67 in COVID-19. A striking increase in K_i_-67^+^ monocytes (<5% in monocytes from most healthy controls) was evident in COVID-19 patients, but was most dramatic in patients with severe disease (Fig. 4, F and G). K_i_-67 expression strongly correlated with CRP levels (Fig. 4H), and with systemic levels of the cytokines IL-6, MCP-1, IP-10 and IL-10 (Fig. S5F), cytokines that were enhanced in COVID-19 patients and tracked with severity (Fig. S2A). Enhancement of K_i_-67 expression was also observed in unstimulated monocytes from COVID-19 patients (Fig. S5G).

We next assessed how monocyte alterations varied over the patients’ hospital stay and noted that patients with mild COVID-19 had consistently higher TNF-α and COX-2 expression in LPS-activated monocytes compared to patients with severe disease (Fig. 4, I and J). Indeed, COX-2 remained low in severe patients throughout intensive care but levels were restored upon recovery in mild patients (Fig. 4L). IL-1β was consistently low over time in both severity groups with no significant differences in monocyte production of IL-1β between the first and last measured time points from mild or severe patients (Fig. S5H). K_i_-67 expression, however, was highest at recruitment and decreased in patients (back down to levels seen in healthy controls) during the progression of disease, independent of severity category or final outcome (Fig. 4, K and L). Thus, defined alterations to monocyte function, specifically to TNF-α and COX-2, are maintained across the disease time-course and levels of expression are associated with severity. Taken together, these findings highlight alterations to monocyte phenotype and function as key features of disease progression and severity in COVID-19.

## DISCUSSION

Respiratory viruses continue to cause devastating global disease. This detailed, prospective, observational analysis of COVID-19 patients of varying severity and outcome, in real time, has revealed specific immunological features that track with disease severity, providing important information concerning pathogenesis that should influence clinical trials and therapeutics. Of particular importance, increased expression of the cell cycle marker K_i_-67 in blood monocytes, reduced expression of COX-2, and a high neutrophil to T cell ratio are early predictors of disease severity that could be used to stratify patients upon admission for therapeutics. Critically, the majority of aberrant immune parameters studied reverted in patients with good outcome. Unexpectedly, multiple aspects of inflammation that were high upon admission, diminished as patients progressed in severity and were admitted to intensive care. In particular, levels of IP-10 and K_i_-67 expression by monocytes were reduced after admission to intensive care, even in patients who did not recover. These data indicate that treating patients early after hospitalization is likely to be most beneficial, while cytokine levels and immune functions are disrupted.

Though other studies have focused on defects in adaptive immunity in COVID-19 pathogenesis (*18*), we demonstrate here considerable abnormalities in the innate immune system, in particular within myeloid cells. Profound neutrophilia exists in severe COVID-19, supportive of a role for neutrophils in acute respiratory distress syndrome (*19*, *20*) and in line with the excess neutrophils seen in the autopsied lungs of patients that died from COVID-19 (*21*). Neutrophils assist in the clearance of pathogens through phagocytosis, oxidative burst and by liberating traps (neutrophil extracellular traps or NETs) that capture pathogens. The latter two functions, however, can also promote inflammation and are associated with many of the features seen in COVID-19 (*22*). Indeed, elevated neutrophil products have been identified in the sera of COVID-19 patients and correlate with clinical parameters such as C-reactive protein, D-dimer, and lactate dehydrogenase (*23*).

Altered monocyte phenotypes were also seen in COVID-19 patients, with patient blood monocytes expressing the cell cycle marker K_i_-67 (up to 98%); a feature not observed in health. This likely represents either early or enhanced release of monocytes from the bone marrow due to systemic inflammatory signals and is similar to that described in pandemic H1N1 influenza (*16*) and Ebola virus infections (*17*). Equally remarkable was the reduced expression of COX-2 in monocytes in patients with severe disease, which was evident across their disease trajectory. COX-2 facilitates the production of prostanoids including prostaglandin E2 (PGE2), and other viruses are known to target this pathway to enhance viral replication (*24*). However, its reduction in monocytes in response to viral lung infection has not previously been reported. Reduced COX-2 alongside high IL-6 and IP-10, as seen here in severe COVID-19 patients, is an immune profile associated with pathology in idiopathic pulmonary fibrosis (IPF) (*25*). Therefore, our data indicate a possible fibrotic signature in patients with severe disease, supporting studies observing an unusual pattern of fibrosis in the lungs of COVID-19 patients.

Our data concur with several features of COVID-19 studied in Wuhan, China, as well as with more recent studies from across the globe (*26*, *27*) and are also corroborated by single cell RNA sequencing of bronchoalveolar lavage cells at a single time point (*28*). Similarities include elevated CRP and IL-6 in patients at the time of hospitalization who eventually died (*29*) and increased IP-10 in those who later developed severe disease (*30*). IP-10 is an interferon-inducible chemokine that facilitates directed migration of many immune cells (*31*) and is elevated in other coronavirus infections including MERS-CoV and SARS-CoV (*32*), as well as in Influenza virus of swine origin (H1N1) (*33*, *34*). The heightened levels of monocyte-chemoattractant protein 1 (MCP-1) upon admission further indicate dysregulation of monocyte function and migration in patients with severe disease. Importantly, IL-6, IP-10 and MCP-1 levels are generally the highest around the time of hospital admission but are reduced rapidly as patients are admitted to intensive care, which may well signify exhaustion of the immune cells producing these mediators.

Examining cells of the adaptive immune system, we identified lymphopenia which is now a well-established hallmark of COVID-19 patients (*35*–*38*). Despite this being a key feature of COVID-19, the drivers of loss of T and B cell numbers in peripheral blood remain obscure and could equally reflect either cell death and/or elevated trafficking to the site of inflammation. Focusing on T cells, the phenotype and function of circulating T cells remain an issue with conflicting reports within the literature. Consistent with previous reports, our data show modest increases in T cell activation (*27*, *39*, *40*), primarily driven by a substantial heterogeneity between patients. Despite this, the frequencies of T cells with activated phenotypes remained stable across the disease trajectory, implying most changes to these adaptive mediators could have occurred prior to hospitalization. Importantly our data highlight activation of a cytotoxic program in CD8^+^ T cells, evidenced by perforin expression, which would support effective viral clearance that has previously been suggested (*41*). Focusing on B cells, patients with severe COVID-19 displayed a dramatic expansion of CD27^–^IgD^–^ double negative (DN) B cells. This is in agreement with a recent study reporting lupus-like hallmarks of extrafollicular B cell activation in critically unwell COVID-19 patients (*42*). DN B cells are also associated with immune senescence as a result of excessive immune activation, and an exhausted phenotype is observed in patients with HIV (*11*). Further studies evaluating the functional capacity of expanded DN B cells will be critical to understand their contribution to severe COVID-19.

There are, of course, limitations to any study of samples during a viral pandemic for which there is no vaccine. However, we believe that these do not diminish the importance of the major findings from our study. A longitudinal analysis in real time for phenotypic, functional and soluble markers naturally limits the number of patients interrogated. In-depth analysis of smaller cohorts however, is necessary to gain insight into mechanism and is of interest to the pharmaceutical industry. It takes time to recruit the appropriate number of control subjects of the approximate gender and age of COVID patients and also with the span of comorbidities associated with the greatest risk from SARS-CoV-2. The majority of our controls were drawn from frontline workers, who produced remarkably similar results to each other. The only other potential limitation is that patients may not accurately define the onset of symptoms. As data are plotted per patient, however, this does not affect the interpretation of the results.

There are clinical implications of our data. Using non-steroidal anti-inflammatory drugs (NSAIDs) remains controversial (*43*) and our study would suggest they may not be desirable, as this may compound the already low COX-2 (*44*). Since most of the pathogenic mechanisms involve myeloid cells, neutrophils and monocytes, it would be advantageous to reduce their influx to the lung once lung pathology is established. Relevant strategies include inhibition of the complement anaphylatoxin C5a (*45*) or IL-8 (CXCL8), which are strong chemoattractants for many immune cells, including neutrophils. Antagonism of CXCR2 that mobilizes neutrophil and monocyte from the bone marrow, neutrophil elastase inhibitors and inhibition of G-CSF, IL-23 and IL-17 that promote neutrophil survival, are also options (*46*). Anti-IL-6, IL-1RA and anti-TNF-α agents are already being investigated for COVID-19 treatment and are relevant to neutrophils, which express the requisite cytokine receptors. Furthermore, JAK inhibitors are currently in clinical trials and may also reduce neutrophil levels (*47*). Targeting toxic products of neutrophils such as S100A1/A2, HMGB1 and free radicals, but also the formation of NETs, could be beneficial (*21*).

In summary, this is a key longitudinal study immune profiling COVID-19 patients that places equal emphasis on innate and adaptive immunity. We identify substantial alterations in the myeloid compartment in COVID-19 patients that have not previously been reported. It would appear that comparable innate immune features have been evident in past pandemics with similar or even different viruses and so focusing immune modulation strategies on neutrophils and monocytes is an urgent priority.

## MATERIALS AND METHODS

### Study design

Between 29^th^ March and 7^th^ May, 2020, adults requiring hospital admission with suspected COVID-19 were recruited from 4 hospitals in the Greater Manchester area. Our research objective was to undertake an observational study to (1) examine the kinetics of the immune response in COVID-19 patients and (2) identify early indicators of disease severity. Informed consent was obtained for each patient. Peripheral blood samples were collected at Manchester University Foundation Trust (MFT), Salford Royal NHS Foundation Trust (SRFT) and Pennine Acute NHS Trust (PAT) under the framework of the Manchester Allergy, Respiratory and Thoracic Surgery (ManARTS) Biobank (study no M2020-88) for MFT or the Northern Care Alliance Research Collection (NCARC) tissue biobank (study no. NCA-009) for SRFT and PAT (REC reference 15/NW/0409 for ManARTS and 18/WA/0368 for NCARC). Clinical information was extracted from written/electronic medical records. Patients were included if they tested positive for SARS-CoV-2 by reverse-transcriptase–polymerase-chain-reaction (RT-PCR) on nasopharyngeal/oropharyngeal swabs or sputum. Patients with negative nasopharyngeal RT-PCR results were also included if there was a high clinical suspicion of COVID-19, the radiological findings supported the diagnosis and there was no other explanation for symptoms. Patients were excluded if an alternative diagnosis was reached, where indeterminate imaging findings were combined with negative SARS-CoV-2 nasopharyngeal (NP) test or there was another confounding acute illness not directly related to COVID-19. The severity of disease was scored each day, based on degree of respiratory failure (Fig. 1B). Patients were not stratified for disease severity if there was no available clinical observation data or patients were recruited more than 7 days after hospital admission. Where severity of disease changed during admission, the highest disease severity score was selected for classification. The first available time point was used for all cross-sectional comparisons between mild, moderate and severe disease. Peripheral blood samples were collected as soon after admission as possible and at 1-2 day intervals thereafter. For longitudinal analysis we elected to correlate clinical data with immune parameters directly, rather than using the WHO ordinal scale on account of the small range of values this affords our inpatient cohort, which our study would not be powered to discern. Healthy blood samples were obtained from frontline workers at Manchester University and NHS Trusts **(**age range 28-69; median age=44.5 years; 42.5% males). Samples from healthy donors were examined alongside patient samples.

### Isolation of PBMCs and serum

Whole venous blood was collected in tubes containing EDTA or serum gel clotting activator (Starstedt). Peripheral blood mononuclear cells (PBMCs) were isolated by density gradient centrifugation using Ficoll-Paque Plus (GE Healthcare) and 50 ml SepMate tubes (STEMCELL technologies) according to the manufacturer’s protocol. Serum was separated by centrifuging serum tubes at 2000 × g at 4°C for 20 min.

### Whole blood lysis

Red blood cell lysis was carried out using 10x volume of distilled water for 10 s followed by addition of 10x PBS to re-establish a 1x PBS solution and stop lysis. Cells were centrifuged at 500 × g for 5 min and lysis repeated if necessary.

### Flow cytometry

White blood cells from lysed whole blood and isolated PBMCs separated by density gradient centrifugation were stained immediately on receipt. The following antibodies were used: BDCA-2 (clone 201A), CCR7 (clone G043H7), CD11b (clone ICRF44), CD11c (clone 3.9 or Bu15), CD123 (clone 6H6), CD14 (clone 63D3), CD16 (clone 3G8), CD19 (clone H1B19), CD24 (clone M1/69 or ML5), CD27 (clone M-T271), CD3 (clone OKT3 or UCHT1), CD38 (clone HIT2), CD4 (clone SK3), CD45 (clone 2D1), CD45RA (clone HI100), CD56 (clone MEM-188), CD62L (clone DREG-56), CD8 (clone SK1), HLA-DR (clone L234), ICOS (clone C398.4A), IgD (clone IA6-2), IgM (clone MHM-88), IgG (clone M1310G05), K_i_-67 (clone K_i_-67 or 11F6), PD-1 (clone EH12.2H7), perforin (clone dG9), CD66b (clone G10F5), CD64 (clone 10.1), IL-1β (clone H1b-98) and TNF-α (clone MAb11), all from Biolegend; and COX-2 (clone AS67) from BD Biosciences. PBMCs were also stimulated in vitro for 3 hours with 10 ng/ml LPS in the presence of 10 μg/ml brefeldin A to allow accumulation and analysis of intracellular proteins by flow cytometry. Cells were cultured in RPMI containing 10% fetal calf serum, L-Glutamine, Non-essential Amino Acids, HEPES and penicillin plus streptomycin (Gibco). For surface stains samples were fixed with BD Cytofix (BD Biosciences) prior to acquisition and for intracellular stains (K_i_-67, COX-2, TNF-α and IL-1β) the Foxp3/Transcription Factor Staining Buffer Set (eBioscience) was used. All samples were acquired on a LSRFortessa flow cytometer (BD Biosciences) and analyzed using FlowJo (TreeStar).

### LEGENDplex

Thirteen different mediators associated with anti-viral responses were measured in serum using LEGENDplex assays (BioLegend, San Diego, USA) according to the manufacturer's instructions.

### Statistics

Results are presented as individual data points with medians. Statistical analysis was performed using Prism 8 Software (GraphPad). Normality tests were performed on all datasets. Groups were compared using an unpaired *t*-test (normal distribution) or Mann-Whitney test (failing normality testing) for healthy individuals versus COVID-19 patients. Paired *t*-test (normal distribution) or Wilcoxon matched-pairs signed rank test (failing normality testing) was used for longitudinal data where first and last time points were examined. One-way ANOVA with Holm-Sidak post-hoc testing (normal distribution) or Kruskal-Wallis test with Dunn’s post-hoc testing (failing normality testing) was used for multiple group comparisons. Correlations were assessed with Pearson correlation coefficient (normal distribution) or Spearman’s rank correlation coefficient test (failing normality testing) for separate parameters within the COVID-19 patient group. Information on tests used is detailed in figure legends. In all cases, a *p*-value of ≤ 0.05 was considered significant. ns, not significant; ^∗^p < 0.05, ^∗∗^p < 0.01, ^∗∗∗^p < 0.001.

## Supplementary Materials

immunology.sciencemag.org/cgi/content/full/5/51/eabd6197/DC1

Figure S1. Immune cell types in COVID-19 patients. Figure S2. Serum cytokines and chemokines in COVID-19 patients. Figure S3. T cell activation in COVID-19 patients. Figure S4. B cell subsets in COVID-19 patients. Figure S5. Monocytes in COVID-19 patients. Table S1. Raw data file (Excel spreadsheet).
